# *Allium hookeri* root extract restores streptozotocin-induced pancreatic β-cells dysfunction in a type 1 diabetic rat model

**DOI:** 10.29219/fnr.v69.12104

**Published:** 2025-10-13

**Authors:** Hyun Ju Kim, Seong-Soo Roh, Sung-Hyen Lee, Miran Kang, Jong-Sik Jin

**Affiliations:** a Kimchi Functionality Research Group, World Institute of Kimchi, Gwangju, Republic of Korea; b College of Korean Medicine, Daegue Haany University, Daegu, Republic of Korea; c Functional Food Division, Department of Agro-food resources, National Institute of Agricultural Sciences, Rural Development Administration, Wanju, Jeonbuk, Republic of Korea; d Kimchi Industry Promotion Division, World Institute of Kimchi, Gwangju, Republic of Korea; e Department of Pharmacy, Jeonbuk National University, Iksan, Republic of Korea; f DLED-Agri-bio Fusion Technology Research Center, Jeonbuk National University, Iksan, Republic of Korea

**Keywords:** Allium hookeri *root*, diabetes, oxidative stress, apoptosis, β-cell regeneration

## Abstract

**Background:**

*Allium hookeri* (AH), a traditional herb in Southeast Asia, has been documented for its significant health benefits in metabolic diseases. This study was to explore the effects of AH root extract (AHRE) on pancreatic β-cell regeneration in streptozotocin (STZ)-induced diabetic rats.

**Methods:**

AHRE (100 mg/kg body weight) was administered daily to STZ-induced diabetic rats for 2 weeks. Serum glucose and insulin levels, total-cholesterol, hemoglobin A1c, alanine transaminase, aspartate transaminase, and pancreatic peroxynitrite and thiobarbituric acid reactive substances were measured. Protein expression associated with pancreatic β-cell apoptosis and regeneration was analyzed through Western blotting.

**Results:**

Diabetic rats exhibited hyperglycemia, insulin deficiency, increased levels of oxidative stress markers, and pancreatic β-cell apoptosis and impairment. AHRE treatment reduced hyperglycemia, insulin insufficiency, and oxidative stress, implying a reduction in pancreatic β-cell apoptosis and restoration of pancreatic β-cell regeneration-associated protein expression.

**Conclusions:**

AHRE can facilitate β-cell regeneration in the impaired pancreatic islets through STZ by reducing oxidative stress markers and apoptosis in pancreatic tissue. Owing to pancreatic β-cells are susceptible to oxidative stress, the protective and enhancing effects of AHRE on the apoptosis and regeneration of these cells may be a significant mechanism for its hypoglycemic effect.

## Popular scientific summary

- STZ-induced rats showed hyperglycemia, insulin deficiency, increased levels of oxidative stress markers, and pancratic b-cell apoptosis.
- AHRE ameliorated b-cell impairement by reducing oxidative stress markers and apotosis and activating b-cell regeneration in pancreatic tissue.

Diabetes mellitus (DM) is a prevalent disease globally, and its incidence is anticipated to increase from 2.8% in 2000 to 4.4% in 2030 (1). DM is featured in hyperglycemia, resulting from dysfunction in insulin synthesis and secretion by pancreatic β-cells and/or impaired insulin sensitivity (2). Chronic hyperglycemia activates numerous metabolic signaling pathways that result in inflammation, cytokine secretion, and cell death, thereby causing diabetic complications (3–5).

Streptozotocin (STZ) is the most extensively used chemical agent for experimental type 1 diabetes mellitus (T1DM) that generates reactive oxygen species (ROS) and impairs glucose oxidation, insulin biosynthesis and secretion, and DNA alkylation, thereby causing pancreatic β-cell dysfunction and apoptosis (6–8). Caspase-3 regulates an apoptotic signaling pathway that is stimulated by oxidative stress, mitochondrial dysfunction, and autophagy, and it is crucial in the pancreatic β-cell apoptosis. B-cell lymphoma-2 (Bcl-2)-associated protein x (Bax) triggers apoptosis by releasing mitochondrial cytochrome c, and Bcl-2 is a primary factor of the antiapoptotic protein (9). β-Cell homeostasis was determined by the balance between proliferation, differentiation, and death under diabetic conditions. Pancreatic-duodenal homeobox-1 (PDX-1) and epidermal growth factor receptor (EGFR) are key transcription factors that regulate pancreatic β-cell differentiation, proliferation, and survival (10, 11). The inhibition of PDX-1 and EGFR signaling in the pancreas by chronic hyperglycemia results in impaired insulin synthesis and secretion and β-cell proliferation (12, 13). Facilitating β-cell proliferation is an effective way to regenerate β-cell in patients with diabetes.

*Allium hookeri* Thwaites (AH) is extensively cultivated and used as medicinal purposes in Southeast Asia (14). Its health benefits are attributed to various phenols, phytosterols, and organosulfur compounds, such as cycloalliin, allicin, and S-allylcysteine (15, 16). AH exhibits antioxidant (17–19), antimicrobial (20), hypoglycemic (21, 22), anti-obesity (23–25), neuroprotective (26, 27), and anti-inflammatory (28) effects. Additionally, it can enhance bone formation (29), gastritis (30), and immune responses (31). In our previous studies, AH root extract (AHRE) protected against pancreatic β-cell injury by downregulating oxidative stress and inflammatory signaling pathways in STZ-induced type 1 diabetic rats (22). AHRE also attenuated LPS-induced inflammatory responses through inhibiting NF-κB activation (28) and upregulated the antioxidant capacity and immune responses in RAW264.7 cells and immune-depressed C57BL/6 mice. AHRE extract showed higher antioxidant activities and immunomodulatory effects than AH leaves (32). Moreover, the hypoglycemic effects of AHRE resulted from the reduction of β-cell compensation and hyperinsulinemia in type 2 diabetic animals and prediabetic subjects (33, 34). The administration of aqueous and ethanol extracts of AHR to type 2 db/db mice decreased fasting glucose, insulin, and glycogen storage levels (35). Overall, substantial evidence has demonstrated the antidiabetic effects of AHRE; however, few studies have focused on its effects on pancreatic islets or β-cell damage in diabetes. Furthermore, the molecular mechanism by which AHRE affects β-cell mass recovery remains unclear. Therefore, this study was designed to investigate the potential role of AHRE on pancreatic β-cell recovery and regeneration in STZ-induced diabetic rats.

## Methods

### Sample

**Fig. 1:**
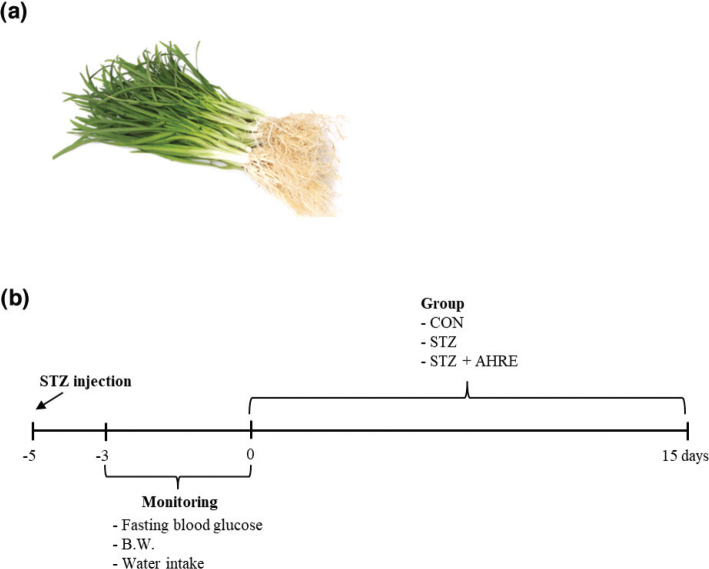
A photograph of the AH (a) and schematic diagram of STZ-induced diabetic study (b).

AH was cultivated and harvested at Sunchang-gun, Jeollabuk-do, Korea, and identified by Dr. Jung Bong Kim (Fig. 1a). The voucher specimen (RDAAH15) was kept in the Functional Evaluation Lab in the Department of Agro-Food Resources, Rural Development Administration (Jeonju, Korea). Extraction method of AHRE was previously described (25). Briefly, dried AHR was extracted in 10 volumes of H_2_O_2_ for 16 h in a water bath at 96°C. The residues were extracted three times under the same conditions. The hot-water extracts were filtered through decompression filtration with Whatman filter paper (grade No. 2; Whatman International, Kent, UK). The extracts were merged and concentrated with a rotary evaporator at 45°C under vacuuming, freeze-dried and kept on storing at −20°C. The yield rate of AHRE was 20.93%.

### Reagents

STZ, phenylmethylsulfonyl fluoride (PMSF), and 2’,7’-dichlorofluorescein diacetate were obtained from Sigma-Aldrich Chemical Co. (St. Louis, MO, USA). Protease inhibitor cocktail and the bicinchoninic acid (BCA) protein assay kit were obtained from Wako Pure Chemical Industries, Ltd. (Osaka, Japan) and Thermo Scientific (Rockford, IL, USA), respectively. Rabbit polyclonal antibodies against Bax, cytochrome c, caspase-3, PDX-1, p-EGFR, and cyclin E, and mouse monoclonal antibody against β-actin, goat anti-rabbit, and goat anti-mouse secondary antibodies were obtained from Cell Signaling Technology (Danvers, MA, USA) and Santa Cruz Biotechnology (Santa Cruz, CA, USA). Enhanced chemiluminescence (ECL) reagents were purchased from GE Healthcare (Piscataway, NJ, USA).

### Animal study

Animal experiments were followed by the Institutional Animal Care and Use Committee (IACUC) of Daegu Haany University (approval no. 2013-036). Fifteen male Sprague-Dawley rats (7-weeks old) were obtained from Daehan-Bio (Chungcheong, Korea) and housed at controlled environmental conditions (temperature [23 ± 2°C] and humidity [50 ± 5%]). In order to induction of diabetes, STZ (45 mg/kg body weight [BW]) in a 10 mM citrate buffer (pH 4.6) was intraperitoneally injected once. To confirm diabetes, serum glucose levels, BW, and water intake were measured after 5 days (36). The successful induction of diabetes was confirmed by fasting plasma glucose level above 250 mg/dL, reduction of BW, and increase of food and water intake, which were monitored for 3 days. The rats were allocated into two groups based on BW and serum glucose levels: diabetic rats that received water (STZ, *n* = 5) and diabetic rats that orally administered 100 mg/kg BW/day of AHRE (*n* = 5) daily. The dose of AHRE was chosen based on an earlier study (22). The non-diabetic group was orally administered distilled water as the normal control (CON, *n* = 5). The photo of AH and schematic diagram of this study were shown in Fig. 1a and b. At 15 days of treatment, rats were fasted for 18 h and anesthetized with injection of pentobarbital (50 mg/kg BW) intraperitoneally (37), and then blood was drawn from the abdominal aorta; the serum was obtained by centrifugation. After euthanasia, the pancreas was harvested and weighted, snap-frozen in liquid nitrogen, and stored at −70°C.

### Biochemical analyses in the serum

Serum glucose (AM201), total-cholesterol (TC, AM202), aspartate aminotransferase (AST, AM103-K), and alanine aminotransferase (ALT, AM102) levels were assessed by Asan kit (Hwaseong-si, Korea), and serum insulin levels (EZRMI-13K) and glycated hemoglobin A1c (HbAlc, RK03710) were assessed by commercial ELISA kit from EMD Millipore (Billerica, MA, USA) and ABclonal (Woburn, MA, USA). Homeostatic Model Assessment for Insulin Resistance (HOMA-IR) was calculated by formula of Matthews et al. (38).

### Assessment of peroxynitrite anion (ONOO-) and thiobarbituric acid reactive substance levels in the pancreas

Pancreatic ONOO^−^ was determined by the rhodamine 123 production method (39). Briefly, sample was added to rhodamine buffer (pH 7.5) and incubated for 10 min at 36.5°C. Optical density at 500 nm was measured for rhodamine 123. Thiobarbituric acid reactive substance (TBARS) concentration was measured by the Buege and Aust (40) method. Briefly, 250 µL of sample was added to 750 µL of 0.4 TBA, 15 TCA (trichloroacetic acid), and 2.5% HCl (hydrochloric acid) solution, boiled at 96~100°C for 15 min. Subsequently, the mixtures were centrifuged at 1,000 × *g* at 25°C for 15 min to transfer the supernatants. The TBARS levels were represented as nM of malondialdehyde per milligram of protein. The protein levels were assessed by the Itzhaki and Gill (41) method.

### Immunoblotting

Western blotting was conducted as described by Jang et al. (28). In brief, pancreatic tissue was homogenized with ice-cold lysis buffer (pH 7.4) containing 137 mM NaCl, 20 mM Tris-HCl, 1% Tween 20, 10% glycerol, 1 mM PMSF, and protease inhibitor mixture. The homogenate was then centrifuged at 2,000 ×g for 10 min at 4°C. The protein concentration was determined using a pierce BCA protein assay kit (A55860, Thermo Fisher Scientific, Rockford, IL, USA). Each 20 μg of protein was electrophoresed with 4–20% sodium dodecylsulfate polyacrylamide gel and transferred to a nitrocellulose membrane. The membranes were cut based on the molecular weights of each protein and incubated with primary antibodies against Bax (#2772), cytochrome c (#4272), caspase-3 (#9662), PDX-1 (#5679), p-EGFR (#3777), cyclin E (#20808) (1:1000; Cell Signaling Technology, Danvers, MA, USA), and β-actin (A5441) (1:10000; Sigma-Aldrich) for 12 h at 4°C. The membranes were rinsed with washing buffer, incubated with secondary anti-mouse or anti-rabbit IgG antibodies for 2 h, and visualized using ECL (Syngene, Frederick, MD, USA). Band densities were normalized with β-actin and quantified using an ImageJ Launcher.

### Statistical analyses

Data are presented as mean ± SEM (standard error of the mean). Significance was determined using a one-way analysis of variance, followed by Dunnett’s multiple comparison test (SPSS, IBM, Chicago, IL, USA). Statistical significance was set at *P* < 0.05. Correlation analysis of type 1 diabetic parameters and proteins related to pancreatic β-cell functions were performed using Spearman’s rank correlation analysis in JMP version 12.

## Results

### Effects of AHRE on body weights and food and water intake

**Fig. 2:**
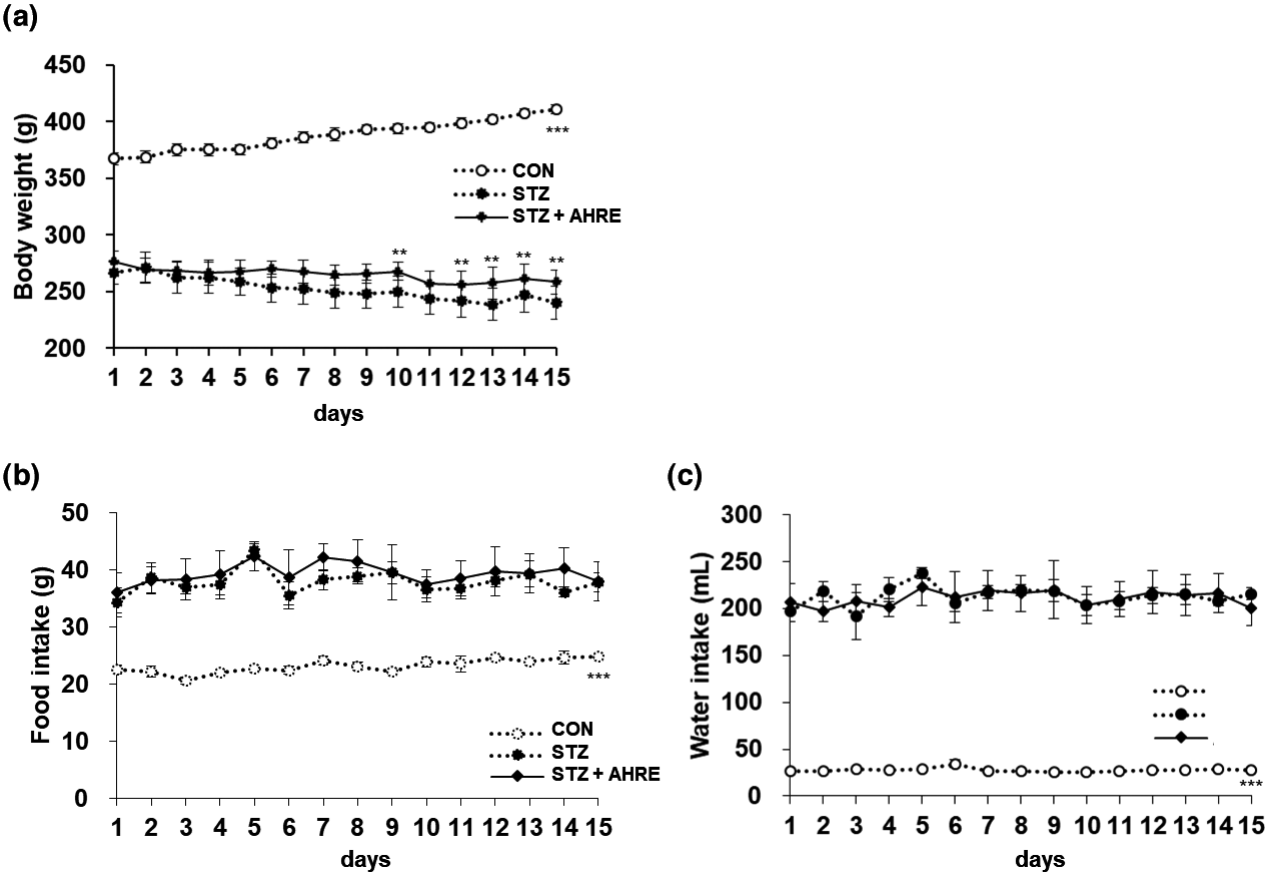
Effects of AHRE on body weight changes, pancreas weight, and food and water intakes in STZ-induced diabetic rats. Body weight (a), food (b), and water intake (c) were measured at every day for the experiment. Data are expressed as mean ± SEM (*n* = 5). **P < 0.01, ***P < 0.001 versus STZ.

Figure 2 illustrates the alterations in BW, and food and water intake during the experiment. BW in the CON group significantly increased by about 40 g/15 days compared to STZ rats, while a marked reduction in BW of the STZ group was recovered by about 2.2 folds through AHRE administration (*P* < 0.001), (Fig. 2a). This implies that AHRE significantly protects the pancreatic β-cell damage and hypertrophy by STZ. The food and water intake was significantly increased by polyphagia and polydipsia but was unaltered through AHRE administration (Fig. 2b, c).

### Effects of AHRE on fasting serum glucose, insulin, HOMA-IR, TC, and HbA1c

**Fig. 3:**
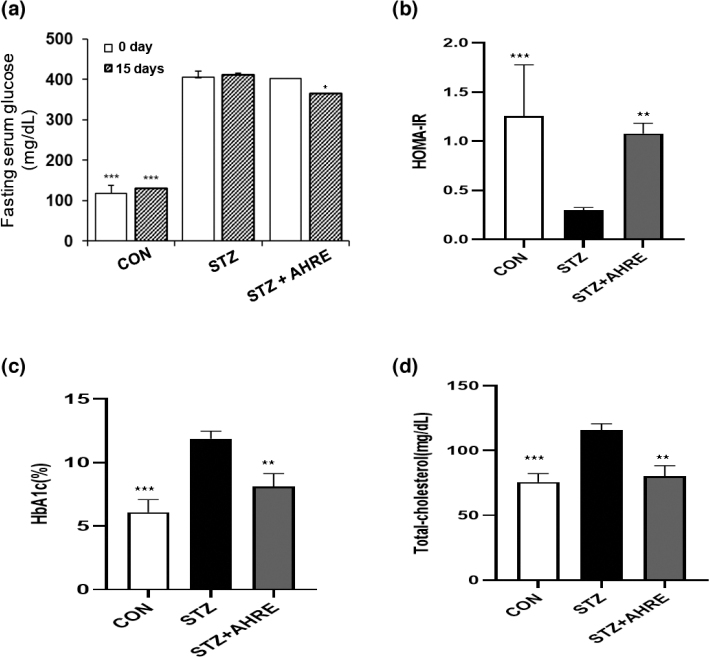
Effects of AHRE on fasting serum glucose (a), HOMA-IR (b), HbAlc (c), and serum total-cholesterol (d) in STZ-induced diabetic rats. Data are expressed as mean ± SEM (*n* = 5). *P < 0.05, **P < 0.01, ***P < 0.001 versus STZ.

As shown in Fig. 3, the STZ rats depicted hyperglycemia (405.9 ± 19.9 mg/dL at 0 day, 411.6 ± 1.6 mg/dL at 15 days) by STZ injection during the experiment period; however, it was significantly reduced by 12% through AHRE administration after 15 days (402.8 ± 15.0 mg/dL at 0 day, 365.7 ± 3.3 mg/dL at 15 days), (Fig. 3a). HOMA-IR, a tool for the assessment of insulin resistance based on fasting insulin and glucose levels, was significantly decreased by about 4.3 folds compared to CON rats, which was significantly recovered by AHRE (Fig. 3b). Increased HbA1c, an indication of the average blood glucose concentration during the preceding 2–3 months, and TC levels by STZ were significantly decreased in AHRE administration (Fig. 3c and d).

### Effects of AHRE on AST and ALT levels

**Fig. 4:**
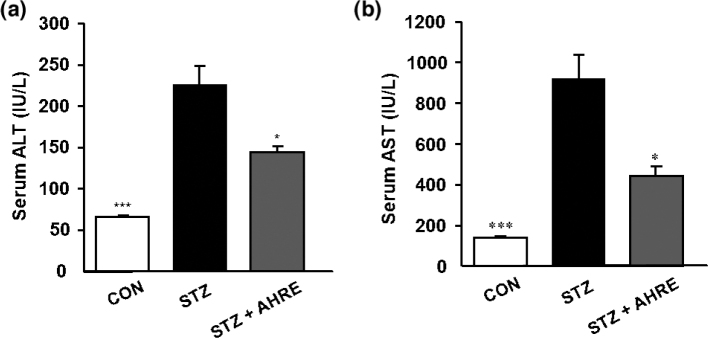
Effects of AHRE on serum ALT (a) and AST (b) in STZ-induced diabetic rats. Data are expressed as mean ± SEM (*n* = 5). *P < 0.05, ***P < 0.001 versus STZ.

The serum ALT and AST levels were significantly higher in the STZ rats by about 3.4 and 6.5 folds than those of the CON rats, respectively, which was significantly reduced by about 1.7 folds and 2.4 folds through AHRE treatment (Fig. 4a and b).

### Effects of AHRE on pancreatic ONOO- and TBARS levels

**Fig. 5:**
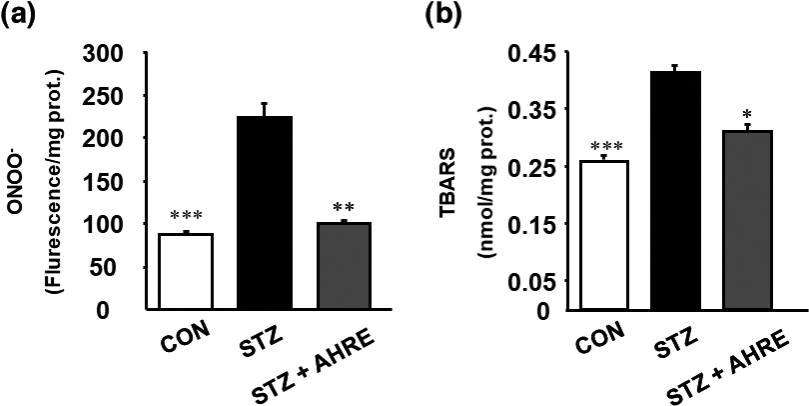
Effects of AHRE on pancreatic ONOO^−^ (a) and TBARS (b) concentrations in STZ-induced diabetic rats. Data are expressed as mean ± SEM (*n* = 5). *P < 0.05, **P < 0.01, ***P < 0.001 versus STZ.

As illustrated in Fig. 5, compared to the CON rats, pancreatic ONOO^−^ and TBARS levels in STZ rats could significantly increase by about 2.4 and 1.5 folds, indicating that STZ releases excessive NO, which destroys pancreatic islet cells through necrosis (7). In contrast, AHRE treatment significantly reduced pancreatic ONOO^−^ and TBARS levels by 2.3 and 1.3 folds, respectively, compared to STZ rats (Fig. 5a and b).

### Effects of AHRE on pancreatic apoptosis-associated protein expression levels

**Fig. 6:**
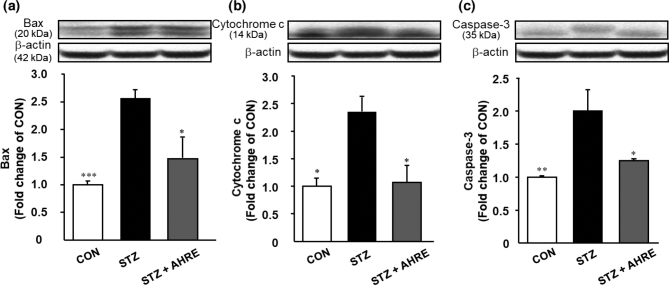
Effects of AHRE on pancreatic protein levels of Bax (a), Cytochrome C (b), and Caspase-3 (c) in STZ-induced diabetic rats. Protein level was determined by Western blot and normalized to β-actin. Data are expressed as mean ± SEM (*n* = 3~5). *P < 0.05, **P < 0.01, ***P < 0.001 versus STZ.

To assess the protective effect of AHRE on apoptosis in the pancreas, the protein expression levels involved in apoptosis were evaluated through western blotting. As illustrated in Fig. 6, the protein expression levels of Bax, cytochrome c, and caspase-3 were significantly increased by about 2.6, 2.4, and 2.1 folds in the pancreatic tissues of STZ rats compared with those in CON rats, respectively. However, AHRE significantly reduced those protein expression by 1.8, 2.2, and 1.8 folds, respectively, to nearly the level in CON rats compared to STZ rats (Fig. 6a–c).

### Effects of AHRE on pancreatic regeneration-associated protein expression levels

**Fig. 7:**
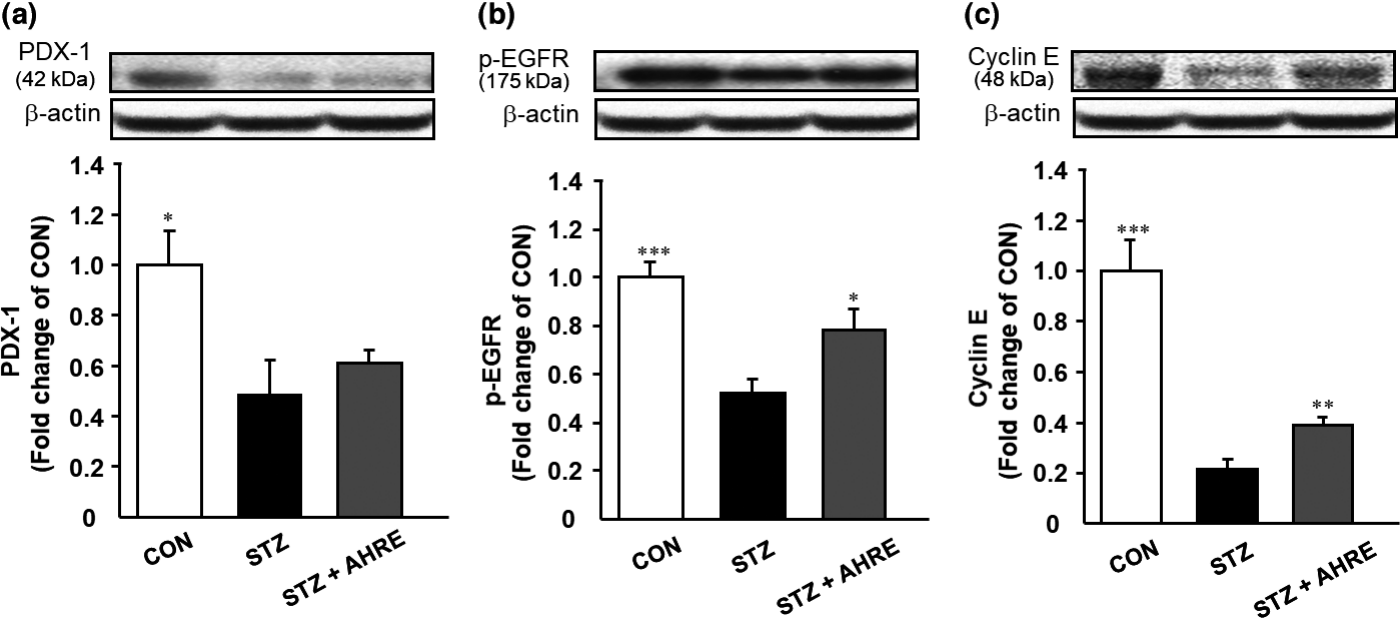
Effects of AHRE on pancreatic protein levels of PDX-1 (a), p-EGFR (b), and Cyclin E (c) in STZ-induced diabetic rats. Protein level was determined by Western blot and normalized to β-actin. Data are expressed as mean ± SEM (*n* = 3~5). *P < 0.05, **P < 0.01, ***P < 0.001 versus STZ.

Based on the results of the aforementioned apoptosis-associated markers, we speculated whether AHRE can affect the pancreatic β-cell regeneration system in STZ rats. As illustrated in Fig. 7, the expression levels of regeneration-associated proteins, such as PDX-1, p-EGFR, and cyclin E, in STZ rats were significantly lower by about 2.1, 1.9, and 4.5 folds than those in the CON rats, respectively. Although PDX-1 protein expression in the pancreas of the AHRE-administered group was not significantly higher by about 1.3 than that in the STZ rats (Fig. 7a), p-EGFR and cyclin E levels were significantly higher by about 1.5 and 1.8 than those in the STZ rats (Fig. 7b and c). This indicates that AHRE may enhance β-cell regeneration by upregulating the protein expression of these transcription factors in the pancreas of STZ rats.

### Correlation analysis between diabetic biochemical parameters and proteins related to pancreatic β-cell functions

**Fig. 8:**
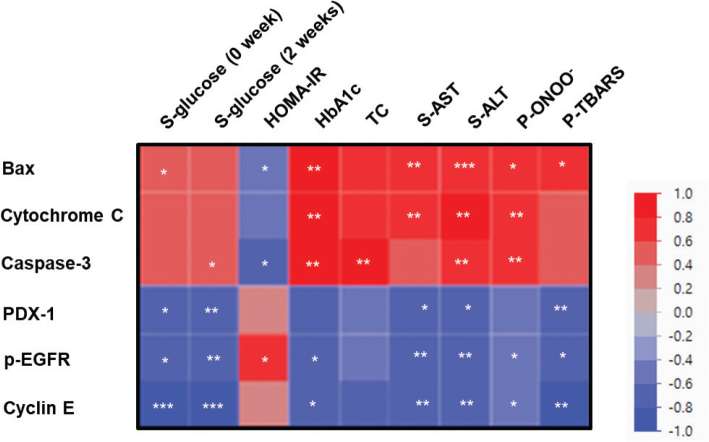
Heat map of the Spearman ‘r’ correlation between the biochemical parameters and proteins related to pancreatic β-cell functions. S-, serum; HOMA-IR, homeostatic model assessment for insulin resistance; HbA1c, glycated hemoglobin A1c, TC, total-cholesterol; AST, aspartate aminotransferase; ALT, alanine aminotransferase; P-., Pancreatic; ONOO^−^, peroxynitrite; TBARS, thiobarbituric acid reactive substance; Bax, Bcl-2-associated protein x; PDX-1, pancreatic-duodenal homeobox-1; p-EGFR, phospho-epidermal growth factor receptor. *P < 0.05, **P < 0.01, ***P < 0.001 following the Spearman correlation.

To ascertain the interaction between diabetic biochemical parameters and proteins related to pancreatic β-cell functions, Spearman analysis was performed (Fig. 8). The fasting serum glucose, liver function, and pancreatic oxidative stress had a positive relationship with protein expression involved in apoptosis and a remarkable negative correlation with protein expression involved in β-cell regeneration, PDX-1, p-EGFR, and cyclin E (*P* < 0.05).

## Discussion

This study aimed to assess alterations in the β-cell function in T1DM condition and clarify the molecular mechanisms regulating the adaptive increase in β-cell regeneration in response to AHRE. Our results demonstrated that AHRE inhibits oxidative stress-induced apoptosis and activates EGFR protein expression that leads to the restoration of β-cell function in the pancreas of STZ-induced diabetic rats. Our previous study showed that AHRE supplementation attenuated hyperglycemia by mitigating the β-cell compensatory response and insulin resistance in *db/db* mice and human subjects with prediabetes (33, 34). This compensatory response is imperative to keep normoglycemia despite insulin resistance, thereby preventing the development and progression of diabetes; however, its underlying mechanisms remain ambiguous. We demonstrated that the protective roles of AHRE on β-cell function in diabetic rats were associated with the inhibition of oxidative stress markers, downregulation of caspase-3, and upregulation of p-EGFR and cyclin E protein expression in the pancreas. As far as we know, this work is the first to assess the effect of AHRE, specifically in pancreatic β-cells. This is supported by our previous findings that AHRE treatment suppressed oxidative stress-induced β-cell damage by downregulating nuclear factor kappa-light-chain-enhancer of activated B cells (NF-κB) signaling pathways in RAW264.7 cells in lipopolysaccharide- and STZ-induced diabetic rats (22, 28).

Overproduction of ROS/reactive nitrogen species by hyperglycemia results in the imbalance of the antioxidant enzyme system, causing pancreatic β-cell apoptosis (42, 43). In addition, O_2_ - can react with NO to produce ONOO^−^, which is a strong oxidizing agent that is significant in the pathogenesis of diabetic complications (44). Moreover, oxidative stress inhibits the activation of transcription factors, such as PDX-1 and V-maf musculoaponeurotic fibrosarcoma oncogene homolog A for β-cell proliferation, differentiation, insulin production, and secretion, consequently resulting in apoptosis under diabetic conditions (45). In our earlier studies, we observed significantly increased levels of oxidative stress and inflammatory markers, as manifested by the decrease of antioxidant enzymes and increase of ROS and NF-κBp65, tumor necrosis factor-α, and interleukin-6 protein expression in STZ-induced diabetic rats, which were reversed through the AHRE administration (22). Consistent with the other studies, we showed that downregulation of PDX-1 by increased oxidative stress was implicated with significantly reduced levels of p-EGFR and cyclin E protein expression and increased levels of apoptotic proteins in the pancreas of T1DM rats (10, 12).

In our previous studies, the primary compounds of AHRE were identified as sulfur compounds, alliin, cycloalliin, and phenolic compound, such as chlorogenic acid and trans-ferulic acid, which were attributed to hypoglycemic effects on diabetes and its associated diseases (22, 32–35).

The non-volatile and volatile organic sulfur compounds from AHR were identified mainly methiin and cycloalliin and allyl sulfides by HPLC-PDA and GC/MS systems, which were attributed to its antioxidant activities, lipid lowering, and antidiabetic effects (19, 46). In addition, allicin content and 10 alkyl thiosulfinates were analyzed in AHR by HPLC-ESI-MS (15). In our previous study, all safety parameters such as AST, ALT, total bilirubin, creatinine, and vital signs were within normal range during AHRE supplementation in prediabetic subjects (33, 34). Our study revealed that increased AST and ALT levels by STZ-induced diabetic condition significantly decreased in the AHRE group, suggesting that there was no adverse effects in liver damage or hepatotoxicity by AHRE. In addition, a new flavonoid compound isolated from the methanolic leaf extract of AH was found to be no toxicity effects and antidiabetic effect by interacting with sulfonylurea receptor 1, which stimulates insulin secretion in pancreatic β-cell of type 2 diabetic rat (21). On the other hand, the investigation has yet to unveil the antidiabetic effects exerted by active compounds after the intake of AHRE in diabetic animal models.

Specifically, in our previous studies, alliin and cycloalliin of AHRE exhibit hypoglycemic effects through pancreatic β-cell protection by reducing oxidative stress, β-cell compensation, and insulin resistance in STZ-induced diabetic rats and patients with prediabetes (22, 33, 34). Moreover, allicin enhances insulin production and pancreatic β-cell function by upregulating the AMP-activated protein kinase/mammalian target of rapamycin-mediated autophagy pathway and inhibits advanced glycation end products by upregulating of antioxidant defense system in the STZ-induced T1DM models (47, 48). In addition, the main flavor of AHR such as diallyl trisulfide, dimethyl trisulfide, dipropyl trisulfide, diallyl disulfides, and methyl allyl is responsible for lowering blood glucose and insulin resistance in high-fat-induced obese mice (49). Garlic extract upregulated genes involved in proliferation and regeneration in the pancreas in T1DM rats (50). Additionally, low-molecular-weight polyphenol consumption reversed pancreatic damage by suppressing apoptosis and increasing proliferation-associated protein expression in T1DM rats (51). In this study, the restoration of p-EGFR and cyclin E protein expression levels through AHRE administration indicated partial recovery of β-cell damage caused by STZ that is corroborated by our previous study, which reported increased β-cell numbers and the normalization of islet structure (22). A reduced PDX-1 expression in T2DM compared with that in non-diabetes because of DNA methylation from hyperglycemia impairs the islet response to insulin sensitivity and exacerbates glucose metabolism (10, 11, 52). Although the PDX-1 protein expression in the pancreas of AHRE-treated rats did not reach significance compared to that in diabetic control rats, AHRE appears to recover the pancreatic β-cell damage and consequently stimulation of insulin secretion. Nilotinib, a potent tyrosine kinase inhibitor member, and polyphenol recover the pancreatic β-cell function through the increase of antioxidant defense system and decrease of oxidative stress as well as the increase of PI3K/Akt/PDX-1 signaling in STZ-induced type 1 diabetic rat models (53, 54). EGFR is predominantly expressed within the islets (55) and is critical for β-cell development, function, and proliferation in diabetes (12, 13, 56). Our previous studies indicated that AHRE may indirectly protect against β-cell impairment by alleviating oxidative stress and preventing overcompensation in the pancreas of patients with T1DM and T2DM (22, 34). This study clearly indicated that AHRE directly inhibited pancreatic damage through the regeneration or proliferation of β-cells, as evidenced by restoring the p-EGFR and cyclin E protein expressions. Consistent with our study, impaired p-EGFR signaling pathway is directly associated with the progression and development of diabetes (12, 13). Contrary to our results, EGFR inhibitor ameliorates the progression of diabetic nephropathy and enhances pancreatic insulin production, resulting in preserved β-cell function and reduced systemic oxidative stress (57, 58).

Cell cycle phases are mediated by various cyclins in a timely and orderly manner. Alterations in cyclin/cyclin-dependent kinases complexes can be responsible for impaired cell growth, thereby delaying tissue repair (59). Due to the well-evidenced significance of PDX-1 and cyclin E in regulating cellular proliferation, we carried out Western blot analysis in pancreatic tissues. In this study, we observed that AHRE can enhance β-cell regeneration, increase insulin secretion, and alleviate hyperglycemia in T1DM rats, accompanied by the upregulation of the protein expression of p-EGFR and cyclin E, which are significant transcription factors in regeneration, development, and maturation of pancreatic β-cells.

A weak point of this study is the absence of standard drugs such as sulfonylureas and meglitinides, which act as a booster of insulin production and secretion in pancreatic β-cells, to compare the hypoglycemic effect of AHRE in the type 1 diabetic rats. Additionally, a confirmation in the dose-dependent effect of AHRE needed to be explored in the future studies. Despite these limitations, this study indicated that the AHRE administration explicitly improved β-cell dysfunction by STZ injection.

## Conclusions

Our results reveal that AHRE exerts a potential protective mechanism on STZ-induced pancreatic β-cell apoptosis and dysfunction in T1DM rats. These effects are associated with the attenuation of hyperglycemia, oxidative stress, and apoptosis and activation of β-cell regeneration. Future studies should clarify the exact mechanisms underlying the hypoglycemic effect of AHRE and its protective effect against β-cell dysfunction in type 2 diabetes models.

## Ethics committee approval

All experimental processes were agreed with the Institutional Animal Care and Use Committee of Daegu Haany University (approval no. 12030) and performed with the Guide for the Care and Use of Laboratory Animals (National Institutes of Health, eighth edition, 2011). All methods were performed in accordance with the relevant guidelines and regulations. This study is reported in accordance with ARRIVE guidelines.

## Consent for publication

Not applicable.

## Data availability statement

All data generated or analyzed during this study are included in this published article.
